# Research on Microstructure Evolution and Rapid Hardening Mechanism of Ultra-Low Carbon Automotive Outer Panel Steel Under Minor Deformation

**DOI:** 10.3390/ma19010128

**Published:** 2025-12-30

**Authors:** Jiandong Guan, Yi Li, Guoming Zhu, Yonglin Kang, Feng Wang, Jun Xu, Meng Xun

**Affiliations:** 1 School of Materials Science and Engineering, University of Science and Technology Beijing, Beijing 100083, China; 2 Beijing Shougang Co., Ltd., Tangshan 064404, China; 3 Shougang Zhixin Electromagnetic Materials (Qian’an) Co., Ltd., Tangshan 064404, China

**Keywords:** steels for automobile outer panels, deformation behavior, microstructure, work hardening, precipitate free zone

## Abstract

With the rapid development of the automotive industry, particularly the year-on-year growth in sales of new energy vehicles, automobile outer panel materials have shown a trend toward high-strength lightweight solutions. Regarding steel for outer panels, existing research has paid less attention to the UF steel that has entered the market in recent years. Moreover, studies on the similarities and differences in deformation behavior among various outer panel steels are lacking. In this study, room-temperature tensile tests at 5% and 8% strain were conducted in accordance with the stamping deformation range on commonly used ultra-low carbon automotive outer panel steels of comparable strength grades, namely, UF340, HC180BD, and DX53D+Z. Prior to deformation, the three materials exhibited similar texture components, predominantly characterized by the γ-fiber texture beneficial for deep drawing, and their room-temperature tensile deformation behaviors were fundamentally identical. After transverse tensile deformation, the textures concentrated towards {111}<112> texture. After 8% deformation, UF340 demonstrated a more rapid stress increase and a higher degree of work hardening. This phenomenon is attributed to the presence of the precipitate free zone (PFZ) near grain boundaries in the UF340, which facilitates the continuous generation of dislocations at grain boundaries during deformation, leading to a rapid increase in dislocation density within the grains. Consequently, this induces accelerated work hardening under small-strain conditions. This mechanism enables UF steels to achieve a strength level comparable to that of bake-hardened (BH) steels, exhibiting a significant performance advantage.

## 1. Introduction

With the rapid development of the automotive industry, especially the annual increase in sales of new energy vehicles (NEVs), the materials used for automotive outer panels are trending toward high strength and lightweight. In particular, the development of NEVs requires reduced overall vehicle weight to achieve longer driving ranges [1,2,3,4]. Adopting higher-strength materials to reduce the thickness of components is a common lightweight method used by automakers. However, the material options for outer covering components are extremely limited. In the traditional automotive industry, the ultra-low carbon steels commonly used for automotive covering components mainly include interstitial free (IF) steel and bake hardening (BH) steel. Due to its relatively low strength, IF steel is difficult to be used as a reinforced material. Zhang et al. from China Automotive Engineering Research Institute [5] and Zhang et al. from Handan Iron and Steel Group [6], respectively, conducted dent resistance tests on typical automotive covering steels, namely, DX53D+Z (IF steel) and HC180BD+Z (BH steel). The results all showed that under the same thickness, HC180BD+Z had better dent resistance than DX53D+Z, and its yield strength was also higher. Whereas, due to the presence of interstitial atoms in BH steel, it has aging issues, and the material also exhibits poor surface smoothness after stamping. Therefore, the automotive manufacturing industry has an increasingly urgent demand for high-strength automotive sheet materials with optimized performance.

To meet market demands, scholars have gradually focused on the research of Nb-IF steel in recent years. The addition of Nb can significantly refine austenite grains, resulting in a fine-grained structure after annealing [7,8]. Meanwhile, by controlling the precipitation and growth of Nb(C, N), the PFZ can be formed near the grain boundaries [9,10]. This not only increases the r-value of the material but also reduces its yield ratio, thereby significantly improving the performance of IF steel. However, all the aforementioned studies remain in the laboratory stage and have not yet achieved industrial production. It was not until 2022 that a type of Nb-added fine-grained high-strength steel for automotive outer panels was developed and named Uni-Fish (UF) steel. This steel grade adopts a combined strengthening mechanism of fine-grain strengthening and precipitation strengthening, featuring high strength and no interstitial atoms. Currently, it has occupied a certain scale of market share [11,12]. Lu et al. compared the mechanical properties and formability of UF340 and HC180BD. They concluded that UF steel of the same strength grade exhibited mechanical properties similar to those of BH steel, and furthermore, UF340 had no obvious bake hardenability, which can avoid aging issues [13].

To date, although some scholars have conducted relevant research on the microstructural evolution and application properties of UF steel [14], there has been no research on the comparison of microstructural and property changes between UF steel and traditional IF steel, as well as BH steel under approximate service conditions. For automotive manufacturers, there exist significant differences in the material selection priorities between automotive outer panels and structural components. The primary function of outer panels is to shape the vehicle’s exterior appearance and styling, and the material deformation range during their stamping process is typically concentrated within a strain range less than 10%. Within this deformation range, the material only needs to meet the forming requirements, without undergoing fracture or bearing substantial external loads during service. Therefore, manufacturers tend to pay less attention to the fracture performance of outer panel steels and instead focus more on their forming behavior under small deformation conditions.

Based on this, this study selects UF340, HC180BD, and DX53D+Z of the same strength grade to conduct room-temperature tensile tests. The microstructural evolution characteristics under the strain range of 5–8% of the three materials are compared in detail, and the rapid hardening mechanism of UF340 under small deformation conditions is investigated systematically, providing theoretical basis and data support for the material selection of steels used in automotive outer panels.

## 2. Experimental Methods

**Figure 1:**
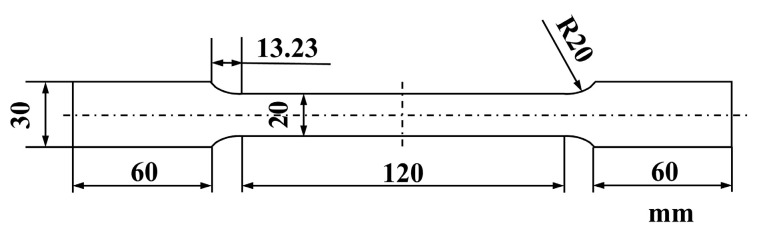
The dimensions of the tensile test samples.

Commercial UF340, HC180BD, and DX53D+Z sheets with a thickness of 0.65 mm and similar strength grades were used in this experiment. Their chemical compositions are listed in Table 1. Room-temperature tensile tests were performed along the rolling direction (RD, 0°), the direction at 45° to the rolling direction, and the transverse direction (TD, 90°) in accordance with GB/T 228.1-2021 [15] using a Zwick Z100 testing machine. The dimensions of the samples are given in Figure 1. The strain rate was set to 0.00025 s^−1^ before yielding, and it was adjusted to 0.0067 s^−1^ by the system once the material yielding was detected. By inputting the plastic strain ratios (r-values) in three directions and the elongation data along the TD with an initial gauge length of 80 mm into the AutoForm software version R13, the forming limit curves (FLCs) of UF340, HC180BD, and DX53D+Z are derived through numerical simulation. The electron back scatter diffraction (EBSD) tests were performed using a Thermo Fisher Apreo 2 S field-emission scanning electron (Thermo Fisher Scientific, Waltham, MA, USA) microscope, with the scanning step size of 0.5 μm. The observation surface of the sample was the rolling direction–normal direction (RD-ND) section at the central position of the tensile specimen. To observe precipitates, some samples of UF340 underwent twin-jet electropolishing using a methanol/nitric acid solution with a ratio 3:1 at a temperature of ~−40 °C and voltage of 15 V, and transmission electron microscopy (TEM) analysis was taken using a JEM-2100 (JEOL Ltd., Tokyo, Japan).

## 3. Results and Discussion

### 3.1. Mechanical Properties

**Figure 2:**
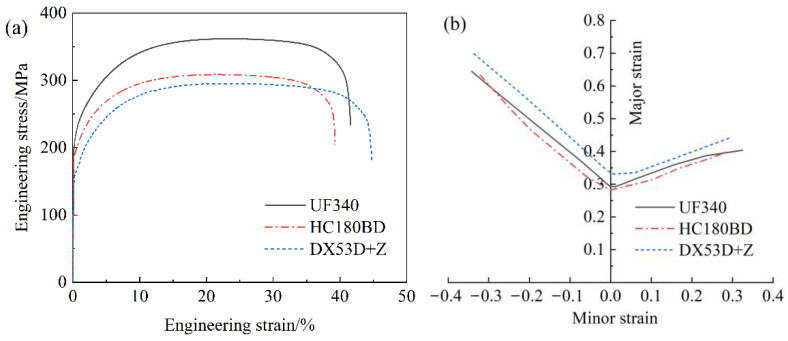
(**a**) Engineering stress–strain curves along the TD and (**b**) FLCs of UF340, HC180BD, and DX53D+Z.

The mechanical properties of the three materials are provided in Table 2. It can be observed that all the three materials exhibit favorable isotropy, with the strength differences at 0°, 45°, and 90° directions within 10 MPa, and the elongation fluctuations within 2%. The tensile stress–strain relations along the TD of UF340, HC180BD, and DX53D+Z are illustrated in Figure 2a. The yield strengths of the three materials are relatively close, but the tensile strength of UF340 is significantly higher than that of HC180BD and DX53D+Z, which indicates that UF340 has the highest degree of work hardening. As can be seen from Table 1, the contents of solid solution strengthening elements, such as Si and P, in UF340 are relatively high, which is consistent with its characteristic of relatively high tensile strength. This characteristic is more favorable for the stamping forming of automotive cover panels. The mechanism underlying this phenomenon will be elaborated in detail later.

The FLCs of the three materials are presented in Figure 2b. It can be observed that the forming limit of DX53D+Z is higher than those of UF340 and HC180BD both in the left uniaxial tension region and the right bulging region, indicating that it possesses superior fracture resistance under these two typical forming conditions and can meet the production requirements of parts with more complex shape. This advantage in formability is highly consistent with the basic mechanical properties of the material, demonstrating that DX53D+Z is more suitable for components where low strength requirements are specified while high formability is demanded. UF340 exhibits intermediate formability, slightly outperforming HC180BH, which indicates a low risk of process instability when switching between the two materials during the stamping process.

For UF340, HC180BD, and DX53D+Z, their main application scenario is the stamping forming of automotive cover panels, with a strain amount ranging from approximately 5% to 8% [16,17]. Therefore, compared with the full-process performance, the mechanical properties and microstructure evolution behavior in the small deformation stage deserve more focused attention. At the strain of 8%, the engineering stresses of UF340, HC180BD, and DX53D+Z are 330 MPa, 284 MPa, and 269 MPa, respectively. Bake hardening (BH), namely, strain aging phenomenon, occurs in the final paint baking process of a car component, and BH steel receives its name from this phenomenon. The strength of BH steel usually can increase by 30–45 MPa after paint baking process. However, the strength of UF340 at the strain of 8% is 46 MPa higher than that of HC180BD, indicating that UF340 can achieve sufficient work hardening effect through conventional stamping deformation. Compared with BH steel, UF steel eliminates the need for the bake hardening process, making it more economical and energy efficient. Moreover, it can avoid material turnover cycle limitation caused by aging. The strength of UF steel with the same thickness is even much higher than that of IF steel, demonstrating excellent dent resistance advantages. Therefore, among the three steel types selected for outer panels, UF steel stands out as the superior choice.

### 3.2. Microstructure

**Figure 3:**
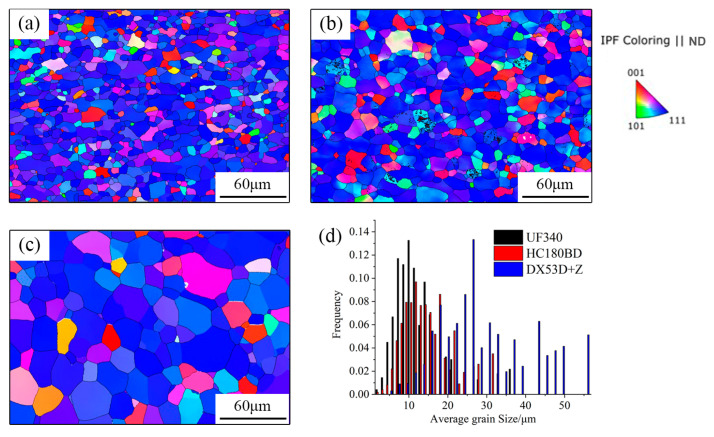
Microstructures of the as-received (**a**) UF340, (**b**) HC180BD, (**c**) DX53D+Z, and (**d**) histogram of grain size distribution.

The inverse pole figure (IPF) maps along the ND of the as-received UF340, HC180BD, and DX53D+Z samples are presented in Figure 3. It can be seen that the three materials exhibit similar microstructures, specifically consisting of equiaxed grains with relatively uniform sizes. UF340 has the smallest grain size, measuring 11.6 μm—approximately 41% of that of DX53D+Z (28.3 μm). HC180BD falls in the middle, with an average grain size of 14.7 μm. Such differences in grain size are mainly attributed to the variations in chemical composition. UF340 contains a relatively high content of Nb. During the production process, the solute Nb retards the recrystallization process, and the formed fine precipitated particles like Nb(C, N) also inhibit grain growth, thereby achieving a grain refinement effect. For IF steel, however, the addition content of alloying elements is relatively low, resulting in a weaker inhibitory effect on grain boundary migration. Furthermore, since IF steel is mostly used for deep-drawing parts, the production process tends to be designed with elevated temperatures to promote more sufficient grain growth, thereby ensuring higher elongation.

Most grains in the three materials exhibit a <111>//ND orientation. The volume fractions of γ-fiber texture in UF340, HC180BD, and DX53D+Z are 73.0%, 61.0%, and 70.9%, respectively. This indicates that all three materials are dominated by γ-fiber texture in the as-received state, ensuring good formability. However, HC180BD has the lowest proportion of γ-fiber texture, which is related to the higher content of interstitial atoms in BH steel. As a result, UF340 and DX53D+Z exhibit better deep-drawing performance than HC180BD.

**Figure 4:**
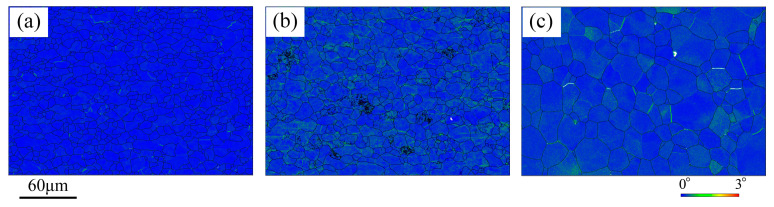
KAM mappings of (**a**) UF340, (**b**) HC180BD, and (**c**) DX53D+Z before deformation.

Figure 4 shows the kernel average misorientation (KAM) maps of the as-received UF340, HC180BD, and DX53D+Z samples. The average KAM values of the three materials are 0.16°, 0.23°, and 0.18°, respectively. UF340 and DX53D+Z exhibit low local misorientation and no obvious dislocation tangles, while HC180BD has a slightly higher KAM value. This is because to ensure the aging stability of BH steel during production, steel manufacturers apply a relatively high skin-pass elongation, leading to a slightly higher degree of work hardening of HC180BD compared to the other two materials.

**Figure 5:**
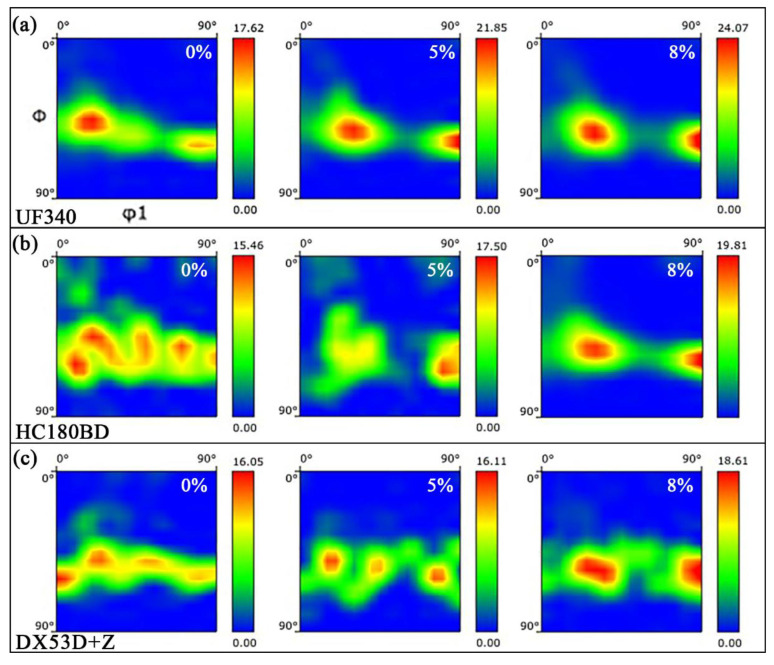
ODF figures of (**a**) UF340, (**b**) HC180BD, and (**c**) DX53D+Z under different deformation conditions.

Room-temperature tensile tests were performed on UF340, HC180BD, and DX53D+Z along the TD. The orientation distribution function (ODF) results at φ2 = 45° for the three materials under different deformation conditions are displayed in Figure 5. The three materials show a certain consistency in their deformation behavior: as the strain increases, the grain orientations gradually concentrate toward {111}<112> texture from a dispersed distribution along the γ-fiber orientation line. Comparing the peak intensities in Figure 4 of the three materials at the strain of 8%, UF340 exhibits a higher intensity, indicating that within the strain range of stamping deformation conditions, the grain rotation in UF steel is more sufficient.

**Figure 6:**
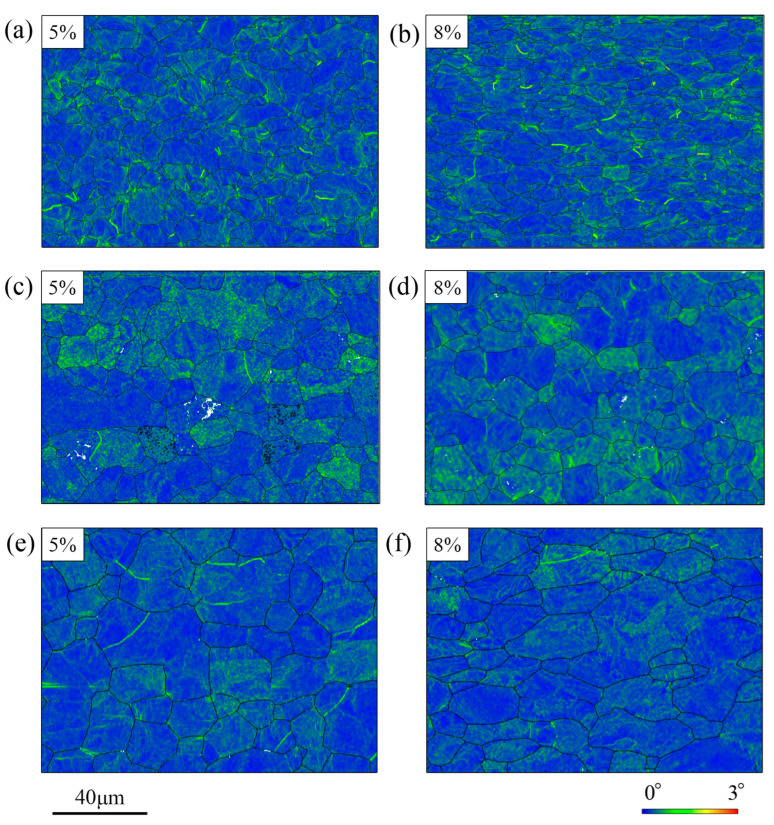
KAM mappings of (**a**,**b**) UF340, (**c**,**d**) HC180BD, and (**e**,**f**) DX53D+Z under 5% and 8% elongation.

Figure 6 exhibits the KAM maps of the three materials at the strain of 5% and 8%. The KAM values of UF340, HC180BD, and DX53D+Z increase from 0.31°, 0.34°, and 0.24°to 0.41°, 0.48°, and 0.38°, respectively. Among the three materials, UF340 has the fastest increase in dislocation density, while DX53D+Z has the slowest, consistent with the slope of the stress–strain curves shown in Figure 1. Notably, deformation in HC180BD and DX53D+Z is concentrated in part of their grains, whereas deformation in UF340 is more uniform, and regions with high KAM values are mostly located near the grain boundaries.

As mentioned before, there are certain differences in grain size among the three materials, with the grain sizes of UF340 and HC180BD being significantly smaller than that of DX53D+Z. Considering that grain size affects the dislocation accommodation capacity, the grain orientation spread (GOS) value (average misorientation within a grain) was normalized by the equivalent circular diameter (D) of the grain (expressed as GOS/D) to evaluate the uniform deformation degree per unit grain length during tensile testing [18,19]. This normalization eliminates the influence of grain size on the average misorientation within grains.

**Figure 7:**
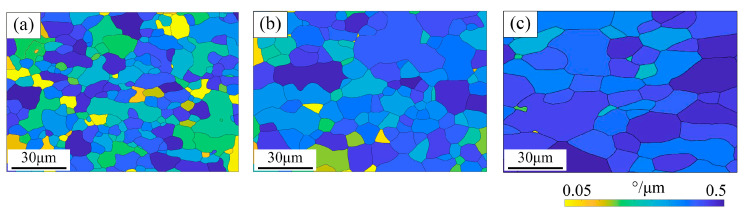
GOS/D mappings of (**a**) UF340, (**b**) HC180BD, and (**c**) DX53D+Z when the tensile deformation is 8%.

The GOS/D comparison results of UF340, HC180BD, and DX53D+Z at the strain of 8% are shown in Figure 7. The average misorientation values per unit grain length of the three materials are 0.272°, 0.243°, and 0.171°, respectively. After normalizing GOS value by grain size, it is clear that UF340 undergoes the largest grain deformation during tensile test, resulting in the highest work hardening degree. DX53D+Z exhibits the lowest strength, coupled with the lowest deformation resistance and the largest grain size; consequently, it shows the lowest GOS/D value. HC180BD contains interstitial atoms that can pin dislocations, along with a lower fraction of γ-fiber texture. As a result, during tensile tests, its deformation tends to be more unevenly distributed, which ultimately leads to an overall work hardening effect that is less pronounced than that of UF340. In other words, when UF steel is used as the outer panel material, and given that the initial strength of the plate is similar with other materials, the strength of UF steel becomes the highest after stamping forming, thus exhibiting better dent resistance.

**Figure 8:**
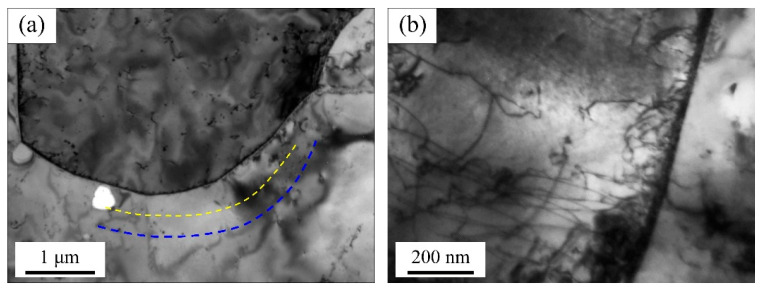
TEM figures of UF340: (**a**) as-received, (**b**) strain of 8%.

Combined with the aforementioned research, UF steel exhibits a higher work hardening degree than the other two materials under the same deformation condition. To clarify the reason for this phenomenon, TEM tests are conducted on UF340 samples before and after tensile test. In the as-received sample, obvious PFZ can be observed on one side of the grain boundaries (as indicated by the blue and yellow dashed lines in Figure 8a). Large precipitates are distributed in a banded pattern in the region approximately 300 nm from one side of the grain boundary, while no precipitates are present adjacent to the grain boundary. In Figure 8b, it can be seen that after tensile deformation, a large number of dislocations are generated at the grain boundaries and pinned by precipitates outside the PFZ.

Due to the presence of PFZ in UF340, the grain boundary regions are in a softened state and tend to yield preferentially during deformation. As a result, dislocation slip first initiates slip at grain boundaries and these dislocations slide out of the PFZ rapidly and accumulate at the precipitate bands in matrix. As strain increases, since no dislocations accumulate in the PFZ, new ones can be continuously generated at grain boundaries. This leads to a rapid rise in the dislocation density within grains, thereby causing a quick increase in strength [20,21]. This is consistent with the KAM map of UF340 in Figure 5a, where a significant stress concentration is observed near the grain boundaries. This phenomenon is precisely caused by the effect of the PFZ.

## 4. Conclusions

In this study, room-temperature tensile tests are conducted on UF340, HC180BD, and DX53D+Z. The microstructural evolution characteristics of the three materials under strains of 5% and 8% are systematically investigated, and the rapid work hardening mechanism of UF340 is analyzed based on TEM and EBSD results. The main conclusions are as follows:

(1) In the as-received state, all three materials consist of equiaxed grains dominated by γ-fiber texture. UF340 has the smallest average grain size and the lowest average KAM value. The tensile deformation behaviors of the three materials are similar. After deformation along the transverse direction, their grain orientations concentrate toward {111}<112>.

(2) After being stretched to the strain of 8%, UF340 shows the fastest stress increase, 46 MPa higher than that of HC180BD, along with the most uniform deformation distribution and the highest work hardening degree.

(3) In UF steel, the precipitate free zone exists near grain boundaries, which causes continuous dislocation generation at grain boundaries during deformation, leading to a rapid increase in dislocation density within grains, and thereby producing a rapid work hardening effect. This makes the strength of UF340 after stamping deformation much higher than that of DX53D+Z and no lower than that of BH steel after bake hardening, exhibiting significant performance advantages.

## Figures and Tables

**Table 1:** Chemical compositions of UF340, HC180BD, and DX53D+Z (wt%).

	C	N	P	Si	Nb	Mn	Ti
UF340	0.0030	0.0014	0.03	0.100	0.045	0.15	-
HC180BD	0.0026	0.0012	0.02	0.004	0.007	0.13	-
DX53D+Z	0.0015	0.0012	0.01	0.004	-	0.15	0.05

**Table 2:** Mechanical properties of UF340, HC180BD, and DX53D+Z.

		Yield Strength/MPa	Tensile Strength/MPa	Elongation/%
0°	UF340	215	357	40
	HC180BD	191	314	40
	DX53D+Z	165	292	45
45°	UF340	217	353	41
	HC180BD	197	317	40
	DX53D+Z	170	296	47
90°	UF340	218	352	41
	HC180BD	194	308	39
	DX53D+Z	168	291	45

## Data Availability

The original contributions presented in this study are included in the article. Further inquiries can be directed to the corresponding author.
